# Factors influencing mental health improvements in school teachers

**DOI:** 10.1371/journal.pone.0206412

**Published:** 2018-10-26

**Authors:** Matthias Braeunig, Ruth Pfeifer, Uwe Schaarschmidt, Claas Lahmann, Joachim Bauer

**Affiliations:** 1 Department of Psychosomatic Medicine and Psychotherapy, Medical Center, University of Freiburg, Freiburg, Germany; 2 COPING Psychologische Diagnostik & Personalentwicklung, Wampersdorf/Vienna, Austria; Centre for Addiction and Mental Health, CANADA

## Abstract

**Objective:**

To identify changes in work-related psychological attitudes that influence mental health improvement in school teachers after participation in a psychological group program.

**Methods:**

In an exploratory study with N = 544 matched cases we combined a screening instrument for general mental health (GHQ) with measures of work-related behavioral and experiential patterns (AVEM). We compared four GHQ change types pre and post intervention with regard to their performance on eleven sub-scales that figure as professional resources. Factors that showed significant relative changes and thus (likely) contributed to improved health status were identified by means of pairwise t-tests and corresponding effect sizes.

**Results:**

Decreases in willingness to work to exhaustion (VB), in striving for perfection (PS), and in the tendency for resignation in the face of failure (RT), as well as an increase of distancing ability (DF) and of inner calm and balance (IR) appear to be the main factors influencing health improvement in the intervention. Simultaneously, an increase of satisfaction with life (LZ) is observed.

**Conclusions:**

The balanced use of professional resources is a critical ingredient in maintaining teachers' health. Adjusting the balance between commitment and resistance through factors found in this analysis help teachers in maintaining and strengthening resilience. The coaching program addresses these factors by focusing on personal attitudes and good interpersonal relationships in the school environment.

## Introduction

The extent to which teachers in Germany and other countries are affected by stress-related health disorders [1], such as depression, anxiety, and somatoform disorders, has not diminished since we first published our results on the effectiveness of our Manual-Based Psychological Group Program for teachers [2,3]. As these results were based on an RCT study, we were appointed by the Baden-Württemberg Ministry of Culture (Kultusministerium) to offer our intervention as part of a state-wide prevention program. The program aims at strengthening health and resilience by fostering competency in relationship-building which includes reflections on stress physiology and work-related attitudes. In the process, we have continually evaluated the outcome of the intervention with different inventories including the General Health Questionnaire (GHQ-12), a screening instrument for mental health we had already applied in our initial study. In this paper we address the important research question that had remained open: Can the observed health benefits of the intervention be correlated with changes in attitudes and coping strategies, and how exactly do they contribute? In order to tackle this question, we applied the AVEM inventory (German for “Arbeitsbezogene Verhaltens- und Erlebensmuster”), an instrument that measures work-related attitudes as professional resources on eleven sub-scales [4]. While other studies that applied AVEM usually focus on the related four risk patterns, e.g. Zimmermann et al. [5] and Voltmer et al. [6], we based our investigation directly on the sub-scales: subjective importance of work (BA), professional ambition (BE), willingness to work to exhaustion (VB), striving for perfection (PS), distancing ability (DF), tendency for resignation in the face of failure (RT), proactive problem-solving (OP), inner calm and balance (IR), experience of success at work (EE), satisfaction with life (LZ), and experience of social support (SU). We investigated whether those who benefited from the intervention, as measured by the GHQ, showed significant higher differences with respect to changes in the AVEM features, compared to those who did not benefit.

## Materials and methods

The research data referred to here are the by-product of the prevention measure that was offered to all school teachers of the region Baden-Württemberg by the state Ministry of Culture (Kultusministerium). The state ministry requested that the intervention should be evaluated by the questionnaires described above. Teachers who decided to respond generated an anonymous code that was later used to match pre and post questionnaires. The data is therefore anonymous and the identity of the respondent is never revealed. As teachers did not undergo physical examination and no biological material was taken, the study did not require approval by the institutional review board (ethics committee). However, with the response of a filled questionnaire written informed consent was given to the use of data for the purpose of evaluation. Since the recorded response was anonymous, the data cannot be traced back to the respondent.

The study included 544 out of 1532 teachers assigned to the program in two consecutive school years (2013/14 and 2014/15). The main reason for this reduction consists in our high standards: Criteria for inclusion were participation in at least five out of six sessions of our intervention or, alternatively, the full-day seminar. Additionally, we only used data from participants who submitted both pre and post questionnaires. Nevertheless the subgroup of the 544 teachers did not differ significantly from the rest in their sociodemographic or GHQ parameters.

The intervention is conceptualized as a Balint-type group work based on a published manual [7]. It includes five modules dealing with the following issues: (1) basic knowledge of stress physiology and the effects on health parameters; (2) mental attitudes with a particular focus on authenticity and identification; (3) competence in handling relationships with students; (4) competence in handling relationships with parents; (5) strengthening collegiality and social support among the staff. Since we have shown that participation in at least five sessions was sufficient for achieving the health benefit [2], the actual program has been shortened from originally ten to currently six sessions. Alternatively, a full- and half-day seminar was offered.

Our analysis was based on data derived from the inventories GHQ-12 [8] and AVEM-44 [9]. Participants were dichotomized with regard to a GHQ cut-off greater than or equal to 4, which indicates an impaired health status [10]. To investigate changes in AVEM parameters in those who improved on the GHQ scale, we divided our sample into four subgroups: (i) “stable healthy” group below the cut-off before and after the intervention; (ii) “improvers” who changed from above to below the cut-off upon intervention; (iii) members of the “worsener” group who changed from below to above the cut-off with the intervention; (iv) “stable at risk” group above the cut-off before and after the intervention. For each group we separately analyzed changes in the means of the eleven AVEM parameters before and after the intervention. Group differences were assessed by paired t-tests, and the corresponding effect sizes calculated, taking into account the correlated design [11]. P-values have been adjusted for multiple testing using Holm-correction [12].

## Results and discussion

**Fig 1:**
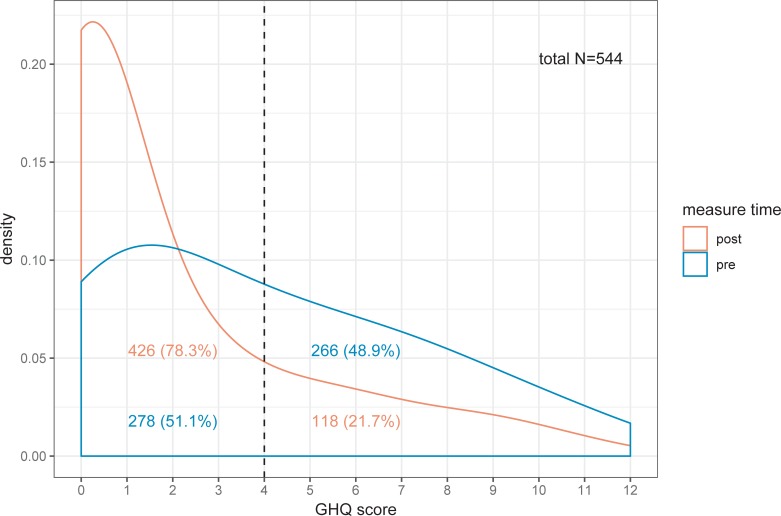
Distribution of GHQ scores before (pre) and after (post) the intervention from a paired sample of participants (N = 544). Note the substantial reduction of impaired GHQ status above cut-off.

**Fig 2:**
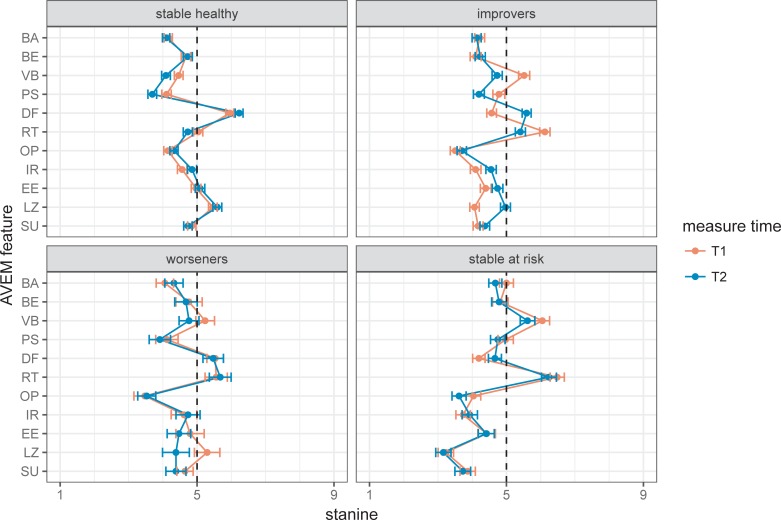
Profiles of AVEM features for each of the four GHQ change types with standard error bars. Acronyms denote subjective importance of work (BA), professional ambition (BE), willingness to work to exhaustion (VB), striving for perfection (PS), distancing ability (DF), tendency for resignation in the face of failure (RT), proactive problem-solving (OP), inner calm and balance (IR), experience of success at work (EE), satisfaction with life (LZ), and experience of social support (SU).

With respect to the GHQ, the sample as a whole improved: As shown in Fig 1, the proportion of those above the cut-off diminished from 49% (266/544) before to 22% (118/544) after the intervention (medium effect size, Cohen’s h = 0.58). However, the focus of this study was not to replicate the effects of the intervention on the GHQ, but to explain them in terms of changed attitudes and coping behaviors as gauged by AVEM features (Fig 2). The following small (d>.2) and medium (d>.5) effect sizes were observed: In the “stable healthy” group PS decreased. For the “improvers” group VB, PS and RT were reduced, while DF, IR, and LZ increased. In the “worsener” group VB and LZ were reduced. In the “stable at risk” groupVB, as well as OP decreased, while DF increased. With respect to the changes with small and medium effects only those in the improvers and “stable healthy” group reached significance (after Holm-adjustment).

Refer to S1 File for a visualization of these results. The relevant factors are shown in Table 1.

**Table 1:** Shortlist of relevant changes in AVEM features.

#	GHQ-type	feature	sig	effect	direction	d	r	df
**1**	stable healthy	PS	***	small	less	-0.20	0.79	239
**2**	improvers	VB	***	small	less	-0.38	0.66	179
**3**	improvers	PS	***	small	less	-0.26	0.73	179
**4**	improvers	DF	***	medium	more	0.56	0.64	179
**5**	improvers	RT	***	small	less	-0.36	0.65	179
**6**	improvers	IR	**	small	more	0.22	0.71	179
**7**	improvers	LZ	***	small	more	0.48	0.60	179

GHQ change type, AVEM feature, statistical significance level (sig), direction, signed effect size (d), correlation (r), degrees of freedom (df). t-test sig. levels: p<0.05 *, p<0.01 **, p<0.001 ***.

Our main finding is that mental health improvements effected by the intervention appear to be influenced by a decrease in willingness to work to exhaustion (VB), in striving for perfection (PS) and in the tendency for resignation (RT), and an increase in distancing ability (DF) and in inner calm and balance (IR). Simultaneously–and probably as a result of these changes–satisfaction with life in general (LZ) increased. Similar albeit smaller AVEM changes could also be observed in the other three GHQ change types. In the “stable at risk” group these changes were obviously not strong enough to result in a positive shift of health status. The “stable healthy” group may have profited from weak but still significant changes in the same direction as in the improvers group, by helping to maintain their already positive health status. The smallest group, the “worseners”, were either not reached by the intervention or had other reasons for their decline.

## Limitations

We are aware that the changes in attitude and experience on health improvement are to be seen as correlates of the four GHQ change types. A deeper understanding of their mediating influence must be gained through further investigation.

The forgoing analysis focuses on effect sizes rather than significance of changes. The effect sizes found are based on the t-test statistic and consider the correlation between pre and post measures of the AVEM features. The p-values are merely used as indicators. However, they have been adjusted by an appropriate adjustment method (Holm-correction) and underline the reliability of the result. The significance level is an aid to support our claim that the effects in the improvers group are to be considered as relevant. Correlation between AVEM features is assumed and the relevant factors may contribute in an orchestrated manner. Nevertheless, an analysis of correlation between AVEM features remains to be done and should be performed in a sequel to this paper.

The missing replacement by median values in T1 and copy in T2 is simple and conservative, and a more sophisticated method could be sought, such as replacement on the item level of the questionnaire. Since missing pertains mainly to the feature `SU`(experience of social support), which is a resource that does not change much under the intervention, we consider our choice of replacement as safe.

## Conclusions

An important lesson to be learned from these results is that teachers’ health can be improved or maintained by intervention programs that promote self-regulation by adjusting commitment and strengthening resistance, i.e. tuning the use of professional resources like the ones that we have outlined above. Our findings are in good agreement with those reported by Roloff Henoch et al [13]. Our intervention explicitly addresses these resources by introducing teachers to specific modules dealing with personal attitudes, focusing on good interpersonal relationships in their school environment and integrating a relaxation technique. Future research should take into account the factors found in this study and model the mental health benefit of intervention strategies accordingly.

## Supporting information

S1 File Electronic supplement. Further information on the analysis conducted in this paper is provided in an electronic supplement, containing extended tables and figures.
(PDF)
