# Supplementary material for: Factors influencing mental health improvements in school teachers
Source: PLoS One. 2018 Oct 26;13(10):e0206412. doi: 10.1371/journal.pone.0206412 (PMC6203395; doi:10.1371/journal.pone.0206412)
Supplement: S1 File — Further information on the analysis conducted in this paper is provided in an electronic supplement, containing extended tables and figures. (PDF) [file pone.0206412.s001.pdf]

Factors influencing mental health improvements in school teachers

S1 Electronic Supplement

M. Braeunig[1], R. Pfeifer[1], U. Schaarschmidt[2], C. Lahmann[1], J. Bauer[1]
[1]Department of Psychosomatic Medicine and Psychotherapy, Medical Center, University of Freiburg, Freiburg, Germany
[2]COPING - Psychologische Diagnostik & Personalentwicklung, Wampersdorf/Vienna, Austria
Corresponding author: prof.joachim.bauer@posteo.de (JB)

Revised version December, 2017

Database

The data set for this analysis is made freely available on the project website (see Data availability).

Two consecutive school years (2013/14 and 2014/15) are considered for this analysis. The teachers have participated in the intervention program "Lehrer-Coachinggruppen nach dem Freiburger Modell" [1,2]. Certain inclusion and exclusion criteria apply (described in the main paper).

Counting cases

Besides the obvious requirements of participation in the intervention as well as matched cases between `T1` and `T2` (refer to main paper), we included into our analysis only complete cases in the GHQ [3], i.e. those who had both observations. Health status is then determined at a cut-off value of greater or equal than 4 on the GHQ scale before and after intervention, giving four possible types for `change` (Table 1).

Table 1: Count of complete cases in GHQ.

GHQ-type	n	proportion
stable healthy	243	44.7%
improvers	183	33.6%
worseners	35	6.4%
stable at risk	83	15.3%

The change below the cut-off is highly significant with the following statistic:

```
    Pearson's Chi-squared test with Yates' continuity correction

data:  cutoff_pre and cutoff_post
```

```
X-squared = 26.64, df = 1, p-value = 2.451e-07
```

Note that it is not the aim of this study to establish the effectiveness of the intervention. So this result only confirms what has been found before in a more rigorous (RCT) fashion [2].

GHQ effect

Effect size for the reduction of impaired GHQ status is calculated from the proportions above cut-off pre and post intervention (using arc-sine approximation [4]):

$$h = |\phi_2 - \phi_1| \ \ where\ \phi_i = 2 \arcsin \sqrt{p_i}$$

Table 2: Measure time (t), GHQ status (cutoff), proportion (prop), arcsin approximation (phi), effect size (h) and effect.

t	cutoff	n	total	prop	phi	h	effect
post	FALSE	426	544	78.3%	2.17	0.58	medium
post	TRUE	118	544	21.7%	0.97	0.58	medium
pre	FALSE	278	544	51.10%	1.59	0.58	medium
pre	TRUE	266	544	48.90%	1.55	0.58	medium

Table 2 shows that the reduction from 266/544=48.90% to 118/544=21.7% of the proportion of impaired health condition corresponds to a medium effect with Cohen's h=0.58.

The reduction is reflected in the GHQ score distribution showing significant changes pre/post intervention (Fig 1).

AVEM parameters

The AVEM inventory [5] has 11 sub-scales listed as follows (Table 3)

Table 3: AVEM features (11 sub-scales) and their abbreviations used in the analysis.

Abbreviation	AVEM.feature
BA	Subjective importance of work
BE	Professional ambition
VB	Willingness to work to exhaustion
PS	Striving for perfection
DF	Distancing ability
RT	Tendency for resignation (in the face of failure)
OP	Proactive problem-solving
IR	Inner calm and balance
EE	Experience of success at work

Abbreviation	AVEM.feature
LZ	Satisfaction with life
SU	Experience of social support

We use stanine (standard-nine) scaled values for the AVEM parameters, normalized to a representative German school teacher population with N=18095 [5].

Replacement for missing values in AVEM parameters

In the GHQ score we required already complete cases, i.e. subjects must have valid GHQ status in pre (`T1`) and post (`T2`) measurement. Eliminating even more subjects, however, for missing values in one or more of the AVEM parameters is too expensive a method of data sanitizing. Therefore, we decided for a conservative missing replacement method, involving two steps: a) missing in pre measure is replaced by the median (most probable value) for that parameter and b) a missing in post measures is replaced by the pre value, thus will stay the same. In 7 cases we found 10 or more parameters missing (i.e. AVEM questionnaire was considered incomplete), those subjects are discarded entirely. The remaining cases have at most 3 missings per data record and are replaced accordingly (Table 4).

Table 4: Cases of missings per data record (subject).

missing	n
0	428
1	73
2	29
3	7
10	1
11	3
12	2
13	1

After replacement there are no missing values left and the database is complete with 537 entries.

Groupwise paired t-tests on AVEM differences

AVEM parameters are measures in pre (T1) and post (T2), paired by anonymous match code. As they are not independent, we perform grouped pair-wise t-tests on the stanine values for each AVEM parameter and over all p-values applied the Holm-adjustment [6] method for multiple testing. The groups are defined by our four types of change in GHQ status, before and after the intervention, labelled as `stable healthy`, `improvers`, `worseners`, and `stable at risk`. GHQ status is defined as logical cut-off condition $ghq \geq 4$, where ghq is the GHQ score on a scale from 0 to 12 (Fig 2).

We are mainly interested in effects and calculate effect sizes (Cohen's d) for correlated samples according to [7] by $d = t_c\sqrt{2(1-r)/n_{df}}$, where t_c is the value of the (paired samples) Student

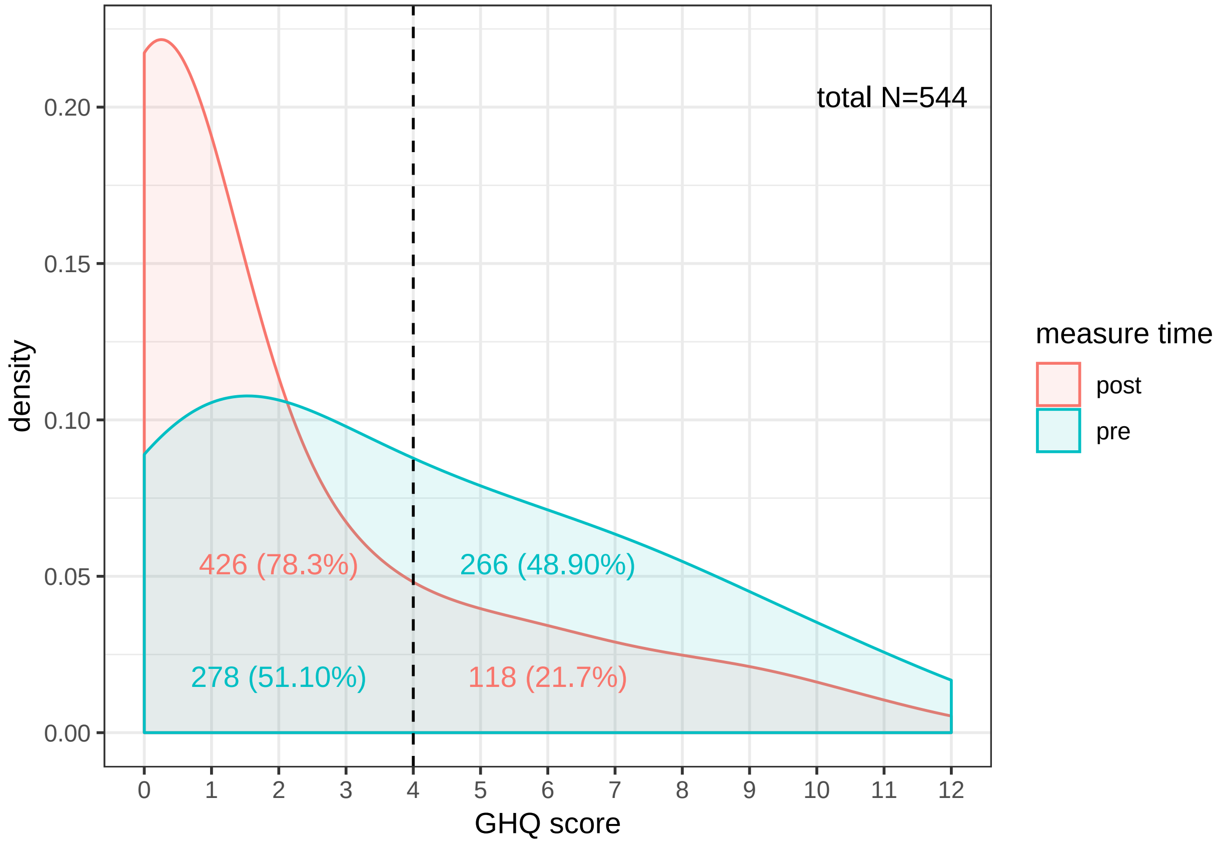

Figure 1: Proportion beyond GHQ cut-off is substantially reduced after the intervention.

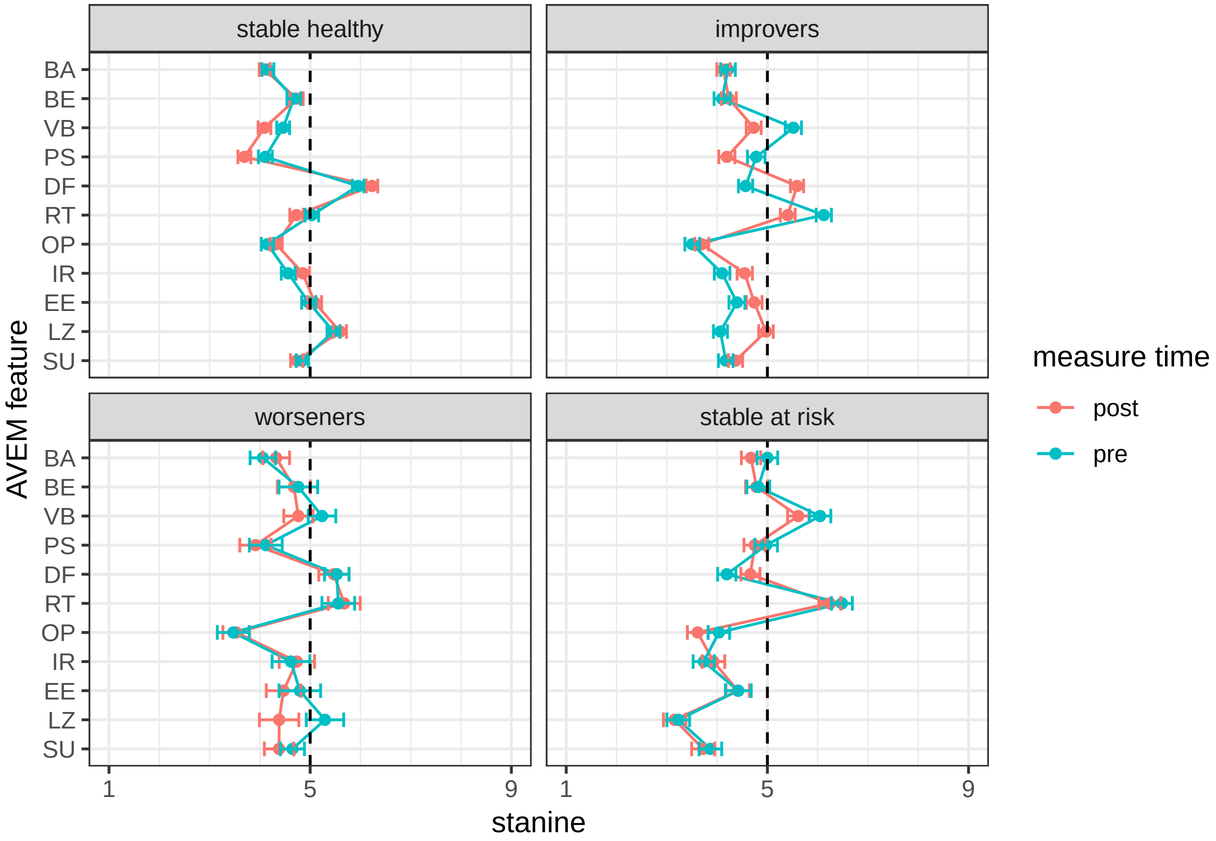

Figure 2: Profiles of AVEM features for GHQ change types with standard error bars.

t-statistic, r is the correlation of the stanine values in each pair of AVEM parameters, and n_{df} is the number of degrees of freedom in the t-test.

Table 5: Complete table of changes in AVEM features, with adjusted significance (sig), effect, direction, effect size (d), correlation (r) and degrees of freedom (df)

	GHQ-type	feature	sig	effect	direction	d	r	df
1	stable healthy	BA			less	-0.04	0.69	239
2	stable healthy	BE			more	0.03	0.80	239
3	stable healthy	VB	**		less	-0.19	0.71	239
4	stable healthy	PS	***	small	less	-0.20	0.79	239
5	stable healthy	DF	.		more	0.16	0.70	239
6	stable healthy	RT			less	-0.15	0.66	239
7	stable healthy	OP			more	0.09	0.66	239
8	stable healthy	IR	.		more	0.14	0.75	239
9	stable healthy	EE			more	0.05	0.76	239
10	stable healthy	LZ			more	0.06	0.73	239
11	stable healthy	SU			less	-0.06	0.74	239
12	improvers	BA			less	-0.05	0.71	179
13	improvers	BE			more	0.06	0.75	179
14	improvers	VB	***	small	less	-0.38	0.66	179
15	improvers	PS	***	small	less	-0.26	0.73	179
16	improvers	DF	***	medium	more	0.56	0.64	179
17	improvers	RT	***	small	less	-0.36	0.65	179
18	improvers	OP			more	0.10	0.68	179
19	improvers	IR	**	small	more	0.22	0.71	179
20	improvers	EE	*		more	0.16	0.79	179
21	improvers	LZ	***	small	more	0.48	0.60	179
22	improvers	SU			more	0.10	0.70	179
23	worseners	BA			more	0.18	0.57	33
24	worseners	BE			less	-0.04	0.81	33
25	worseners	VB		small	less	-0.29	0.74	33
26	worseners	PS			less	-0.11	0.82	33
27	worseners	DF			less	-0.04	0.61	33
28	worseners	RT			more	0.06	0.69	33
29	worseners	OP			more	0.03	0.54	33
30	worseners	IR			more	0.06	0.80	33
31	worseners	EE			less	-0.14	0.73	33
32	worseners	LZ		small	less	-0.42	0.64	33
33	worseners	SU			less	-0.17	0.60	33
34	stable at risk	BA			less	-0.18	0.80	82
35	stable at risk	BE			less	-0.02	0.70	82
36	stable at risk	VB		small	less	-0.22	0.73	82
37	stable at risk	PS			less	-0.12	0.69	82
38	stable at risk	DF		small	more	0.28	0.62	82
39	stable at risk	RT			less	-0.12	0.59	82

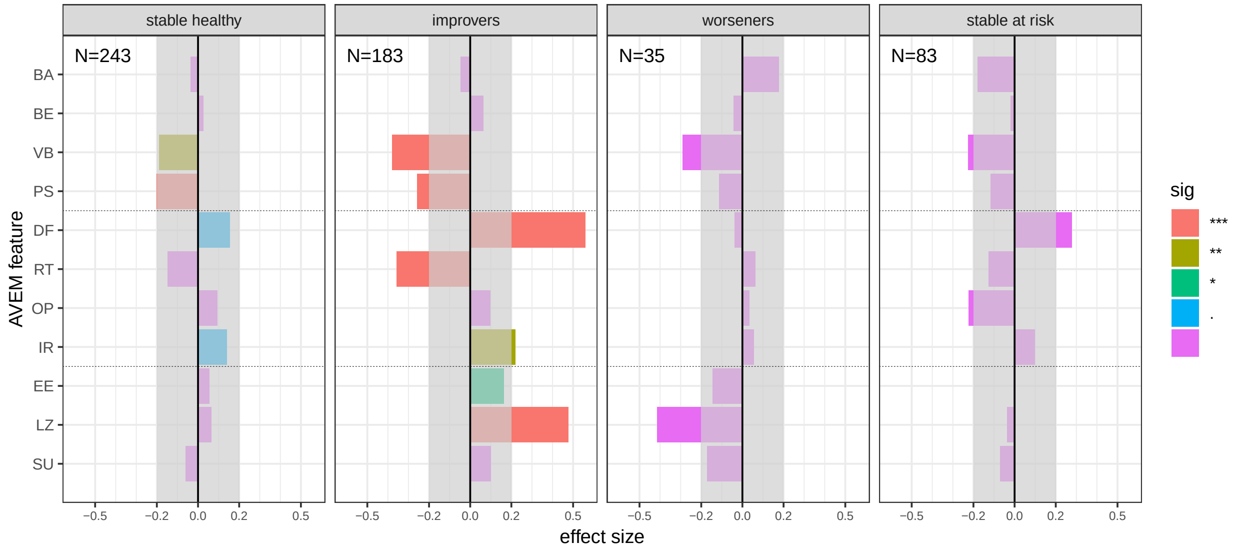

Figure 3: Effect size of changes in AVEM features for GHQ change types (from two-sided paired t-tests).

	GHQ-type	feature	sig	effect	direction	d	r	df
40	stable at risk	OP		small	less	-0.22	0.70	82
41	stable at risk	IR			more	0.10	0.76	82
42	stable at risk	EE			less	-0.01	0.77	82
43	stable at risk	LZ			less	-0.04	0.78	82
44	stable at risk	SU			less	-0.07	0.77	82

As can be seen in Fig 3, highly significant effects only show in the improvers type, whereas in the other types effects are either too small or insignificant. In the worseners type it can be argued that the decrease in `VB` (the willingness to work to exhaustion) is not a result of the intervention but a consequence of exhaustion itself. The change in AVEM feature `LZ` (satisfaction with life) in general is a consequence of health improvement or decline.

Limitations

We are aware that the changes in attitude and experience on health improvement are to be seen as correlates of the four GHQ change types. A deeper understanding of their mediating influence must be gained through further investigation.
The forgoing analysis focuses on effect sizes rather than significance of changes. The effect sizes found are based on the t-test statistic and consider the correlation between pre and post measures of the AVEM features. The p-values are merely used as indicators. However, they have been adjusted by an appropriate adjustment method (Holm-correction) and underline the reliability of the result. The significance level is an aid to support our claim that the effects in the improvers group are to be considered as relevant. Correlation between AVEM features is assumed and the relevant factors may contribute in an orchestrated manner. Nevertheless, an analysis of correlation between AVEM

features remains to be done and should be performed in a sequel to this paper.
The missing replacement by median values in T1 and copy in T2 is simple and conservative, and a more sophisticated method could be sought, such as replacement on the item level of the questionnaire. Since missing pertains mainly to the feature `SU` (experience of social support), which is a resource that does not change much under the intervention, we consider our choice of replacement as safe.

Acknowledgement

The health promotion program "Lehrer-Coachinggruppen nach dem Freiburger Modell" (https://lehrer-coachinggruppen.de) is supported by the Kultusministerium Baden-Württemberg. The authors are grateful to Marieluise Bethke and to Dr. Tobias Walker for their long-standing cooperation in the context of this project.

The article processing charge was funded by the German Research Foundation (DFG) and the University of Freiburg in the funding programme Open Access Publishing.

This paper relies heavily on principles of reproducible research performed with R version 3.4.4 (2018-03-15) [8] using RStudio daily built version 1.2.227 [9] and the 'tidyverse' [10].

Data availability

The data set is made freely available at the Open Science Framework (OSF) public repository at https://osf.io/ns74z/ (DOI 10.17605/OSF.IO/NS74Z).

Note: The file `GHQ_AVEM.data.rds` includes a minimal data set of which the above analysis can be reproduced. The data can be restored with the `R`-function `readRDS`.

References

1. Bauer J. Handlungsanleitungen zur Erhaltung und Förderung der Gesundheit von Lehrerinnen und Lehrern. [Internet]. Dresden: Techn. Univ. 2007. Available: https://lehrer-coachinggruppen.de/manual.pdf

2. Unterbrink T, Zimmermann L, Pfeifer R, Rose U, Joos A, Hartmann A, et al. Improvement in School Teachers' Mental Health by a Manual-Based Psychological Group Program. Psychotherapy and Psychosomatics. 2010;79: 262–264. doi:10.1159/000315133

3. Goldberg DP, Williams P. A User's Guide to the General Health Questionnaire. Windsor: NFER-Nelson; 1988.

4. Cohen J. Statistical power analysis for the behavioral sciences [Internet]. 2nd ed. Hillsdale, N.J: L. Erlbaum Associates; 1988. Available: http://www.sciencedirect.com/science/book/9780121790608

5. Schaarschmidt U, Fischer AW. AVEM Work-related Behavior and Experience Patterns [Internet]. 2008. Available: https://www.schuhfried.com/test/AVEM

6. Holm S. A Simple Sequentially Rejective Multiple Test Procedure. Scandinavian Journal of Statistics. 1979;6: 65–70. Available: http://www.jstor.org/stable/4615733

7. Dunlap WP, Cortina JM, Vaslow JB, Burke MJ. Meta-analysis of experiments with matched groups or repeated measures designs. Psychological Methods. 1996;1: 170. Available: http://psycnet.apa.org/journals/met/1/2/170/

8. R Core Team. R: A Language and Environment for Statistical Computing [Internet]. Vienna, Austria: R Foundation for Statistical Computing; 2016. Available: https://www.R-project.org/

9. RStudio Team. RStudio: Integrated Development Environment for R [Internet]. Boston, MA: RStudio, Inc. 2015. Available: http://www.rstudio.com/

10. Wickham H. Tidyverse: Easily Install and Load the 'Tidyverse' [Internet]. 2017. Available: https://CRAN.R-project.org/package=tidyverse
